# Supplementary material for: PCR-Based Microarray Enhances Diagnosis of Culture-Negative Biopsied Tissue in Patients with Invasive Mold Infections: Real-World Experience in a Tertiary Medical Center
Source: J Fungi (Basel). 2024 Jul 29;10(8):530. doi: 10.3390/jof10080530 (PMC11355750; doi:10.3390/jof10080530)
Supplement: Supplementary file 1 [file jof-10-00530-s001.zip › Supplementary Figure S1.pdf]

Supplementary Figure S1. Arrangement of mold species-specific oligonucleotide probes on the microarray chip.

	1	2	3	4	5	6	7	8	9	10	11	12
A	Abcor3	Acfal1ax2	Acfus2a+Acfus2b	Ackil2-1	Ackil3-1	M	Acstr5	Alalt3-2	Ascla3	Asfla4	Asfum2a	Asnid2
B	Asnig2	Aster2-t	Asver4-5t	Aupul2	Aupul3	M	Aupul4	Bebas5-t	Blder2-t	Chfun2-2	Chglo1	Clban5
C	Clcar2	Ccla2-2	Corec2-8	Cuber3	Cun4	M	Curt	Cuspi3	Epflo2c-2	Exder1-t	Exjea-t	Exwer
D	Focp	Fopin1	Fumon-t	Fumop	Fusol2a-4	M	Gecan	Gecap3	Mafil4	Miaud3b	Mican2gx2-3	Micok4
E	M	M	M	M	M	NC	M	M	M	M	M	M
F	Mifer1c	Migal3	Migyp3b	Migyp5b	Minan1	M	Miper2	Mrac2-1	Mrac3-1	Pabra2-t	Palil4	PC
G	Pajav	Pavar2	Pbre1-1	Pchr1-1	Pihor2-t	M	Pcor1-2R	Pcor2-2R	Pemar2	Phric3-t	Phver3	Psboy3
H	Ripu4	Riory4	Rsto4	Scbre3-t	Sccha4-5	M	Scpro4-1	Sydh-t	Scha1-4	Taeme4	Taeme6	Trmen2d
I	Trmen3t x2-1	Trrub3-5	Trrs1c	Trsch2b	Trter1	M	Trter6b	Trton1e	Trver2e	Trvio1c	Tvir2-1	Ulcon2-3

PC, positive control. NC, negative control. M, position marker.
