# Supplementary material for: PCR-Based Microarray Enhances Diagnosis of Culture-Negative Biopsied Tissue in Patients with Invasive Mold Infections: Real-World Experience in a Tertiary Medical Center
Source: J Fungi (Basel). 2024 Jul 29;10(8):530. doi: 10.3390/jof10080530 (PMC11355750; doi:10.3390/jof10080530)

Supplementary Figure S2. Processes of oligonucleotide hybridization with specific probes in the DNA microarray.

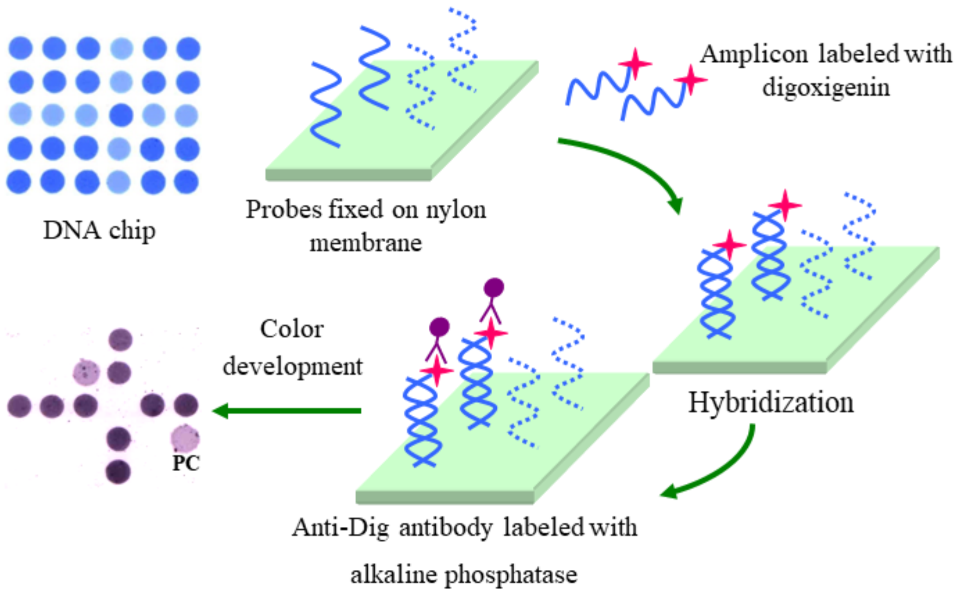

Supplement: Supplementary file 1 [file jof-10-00530-s001.zip › Supplementary Figure S2.pdf]
