# Supplementary material for: PCR-Based Microarray Enhances Diagnosis of Culture-Negative Biopsied Tissue in Patients with Invasive Mold Infections: Real-World Experience in a Tertiary Medical Center
Source: J Fungi (Basel). 2024 Jul 29;10(8):530. doi: 10.3390/jof10080530 (PMC11355750; doi:10.3390/jof10080530)
Supplement: Supplementary file 1 [file jof-10-00530-s001.zip › Supplementary Table S1.pdf]

Supplementary Table S1. Molds and corresponding probe codes.

Target microorganism	Probe	
	Code	Sequence
Absidia corymbifera	Abcor3	CTGGGCTTCTAGTTGATGGCATTTAGTTGC
Acremonium (Fusarium) falciforme	Acfal1ax2	GGAAGCCCCCTGCGGGCACAACGCTTTGGAAGCCCCCTGCGGGCACAACGCttttttttt
*Acremonium kiliense**	Ackil2-1	TCTGATTTTATTGTGAATCTCTGAGGGGCGttttttttt
	Ackil3-1	TGATTTTATTGTGAATCTCTGAGGGGCGAAttttttttt
Acremonium strictum	Acstr5	TTCCGGGGGAGCGGGCCTGttttttttt
*Acrophialophora fusispora**	Acfus2a	CCGTTAAAAGCCTCYAAATAACCCttttttt
	Acfus2b	CCGTTAAAAGCCTCYAATACCCttttttt
Alternaria alternata	Alalt3-2	CGCACTCTCTATCAGCAAAGGTCTAGCA
Aspergillus clavatus	Ascla3	CGACACCAACCCAATYTTTCTAAGGT
Aspergillus flavus	Asfla4	CGAACGCAAATCAATCTTTTTCCAGGT
Aspergillus fumigatus	Asfum2a	GCCAGCCGACACCCAACTTTATTTTTCTAAGtttttt
Aspergillus nidulans	Asnid2	GCGTCTCCAACCTTATTTTTCTCAGGT
Aspergillus niger	Asnig2	ACGTTTTCCAACCATTCTTTCCAGGT
Aspergillus terreus	Aster2-t	CCGACGCATTTWTTGTGCAACTTGTTTttttttttt
Aspergillus versicolour	Asver4-5t	ACGTCTCCAACCATTTTCTTCAGGTttttt
*Aureobasidium pullulans**	Aupul2	ATTTCTAACAACGCTCTTTGGGTCGGTACG
	Aupul3	TCAAAGGAGAGGACTTCTGCCGACTGAAAC
	Aupul4	GGCGTAGTAGAATTTATTCGAACGTCTGTC
Beauveria bassiana	Bebas5-t	GGGACCTCAAACTCTTGTATTCCAGCATCttttttttt
Bipolaris(*Curvularia*) *spicifera*	Cuspi3	GCCTTAAAATGATTGGCAttttttttt
Blastomyces dermatitidis	Blder2-t	CCGCTAGAACTTCTGGTGAACGATTGACATttttttttt
Chaetomium globosum	Chglo1	CTCGCTCTGGTCGCGCCGCttttttttt

Chaetomium funicola	Chfun2-2	CGTAGTAGCATATCTTTGTCTCGCTCAGGtttt
Cladophialophora bantiana	Clban5	TCTTCTCCCTCATGTGGGAAACATTGCA
Cladophialophora carrionii	Clcar2	AGGCCACGGTCCTCTCCTCTAA
Cladosporium cladosporioides	Ccla2-2	CGGGAGGCTACGCCGTAAAtttttttttt
Cokeromyces recurvatus	Corec2-8	TTTAGACTTTGAGGCAGCCCAAATTATACTtttttttttt
Cunninghamella bertholletiae	Cuber3	CACTCTCGGCCTAAATATAAGGCTCGAC
Cunninghamella spp.	Cun4	GAGATAAATTATTACTGGTCCTGGTGATTC
Curvularia spp.	Curt	TCTTTGGCCCGCCAAAGACTCGCCTTAAAtttttttttt
Epidermophyton floccosum	Epflo2-2	TCCATAGGTGGTTCAGTCTGATCGTT
Exophiala dermatitidis	Exder1-t	ACTCTTGAATCAAATCGTGTCCAATGTCTGtttttttttt
Exophiala jeanselmei	Exjea-t	CCAAACGTGTCTTGTCTGAGTAAACGTCtttttttttt
Exophiala werneckii (Hortaea werneckii)	**Exwer**	GTCCGTCTCTAAGCGTTGTGAATAGCGATC
*Fonsecaea compacta/F. pedrosoi***	Focp	CTCACGGGAACACTTTTTTTTTTAAGGT
Fusarium moniliforme	Fumon-t	CGAGTCAAATCGCGTTCCCCAAATTGtttttttttt
*Fusarium moniliforme/F. oxysporum/F. pallidoroseum***	Fumop	AGTAGTAAAACCCTCGTTACTGGTAATCGT
Fusarium solani	Fusol2a-4	ACCTCGCGACTGGAGAtttttttttt
Fomitopsis pinicola	Fopin1	CAATCGTCCTTCACGGGACAATAACTT
Geotrichum candidum	Gecan	CTCTCTTGGAATTGCWTTGCTYTTCTAAA
Geotrichum capitatum	Gecap3	GTGCAACAAGCTGTGTTGAATCTTTC
Malbranchea filamentosa	Mafil4	CGGCGCTGGTCAGAACCAAATCTTTTA
Microsporum audouinii	Miaud3b	CGACCGTCCCCCCCCAATAACtttttttttttt
Microsporum canis	Mican2gx2-3	AGTAACCACCCACCTTTTCCAACTCCCCAGTAACCACCCACCtttttttttt
Microsporum cookei	Micok4	GCCTTGACTGGACTCCTTTGTCCGTTAAAT

Microsporum ferrugineum	Mifer1c	CCCCTCCCCAACAACCACCCACttttttt
Microsporum gallinae	Migal3	GGCCTCGTTTCAATAATTGTCGTTAGAGAAT
*Microsporum gypseum**	Migyp3b	CGGTTTTCTGGCCTAGTTTTAGTTAGG
	Migyp5b	CCTAGTTTCCGTCAGAGATGTATTT
Microsporum nanum	Minan1	AGGAACGATCAAAACACGCGAACAC
Microsporum persicolour	Miper2	TTTTTTTTTGGCCTAGTTTTGGTCAAGGAC
*Mucor racemosus**	Mrac2-1	GGGCCTCTCGATCTGTATAGATCTTttttttttt
	Mrac3-1	TAGATCTTGAAATCCCTGAAATTTACTttttttttt
Paecilomyces javanicuss	Pajav	TAGTACTCCAACGCGCACCGGGAA
Paecilomyces lilacinus	Palil4	GAAATGCAGTGGCGACCYCGCttttttt
Paecilomyces variotii	Pavar2	CCGAAGACCCCTSGAACGCttttttttt
Paracoccidioides brasiliensis	Pabra2-t	TTCGGAGCTTTGACGTCTGAGACCTATCATttttttttt
Penicillium brevicompactum	Pbre1-1	ACCCGCTTTGTAGGACTGCCCGttttttttt
Penicillium chrysogenum	Pchr1-1	TCAACCCAAATTTTTATCCAGGttttttttt
*Penicillium corylophilum**	Pcor1-2R	CGCGGGCCAGAGGGCAGAttttttttttt
	Pcor2-2R	CGCGGGCCAGAGGGCAGAAGttttttttttt
Penicillium marneffei	Pemar2	GTCACCACCATATTTACCACGGTT
Phialophora richardsiae	Phric3-t	CCACTAAAACTCTTCTGTATCTCGCGTACCttttttttt
Phialophora verrucosa	Phver3	CCAGGACCCGGTCCTTCTCCTTTAAC
Piedriaia hortai var. *hortai*	Pihor2-t	AAACGATCCGCCCAGCGAGAATttttttttt
Pseudallescheria boydii	Psboy3	GGTTGCCTTCTGCGTAGTAAGTCTCTTTTG
Rhizomucor pusillus	Ripu4	ATCCGTTCAAGCTACCCGAACAATTTGTAT
Rhizopus oryzae	Riory4	GCAGGAATATTACGCTGGTCTCAGGATCTT
Rhizopus stolonifer	Rsto4	AAAGGCGGTTAATGGTATCCAACAAATttttttttt
Scopulariopsis brevicaulis	Scbre3-t	TGCGTAGTAGATCCTACATCTCGCATCGttttttttt

Scopulariopsis chartarum	Sccha4-5	AGTAAAGCACCTCGCATCGGATCCtttttttttt
Scedosporium prolificans	Scpro4-1	CCAGCCGTCAAACCCTCTATTCTTAT
*Scytalidium dimidiatum/S. hyalinum***	Sydh-t	AACTCCGGTCAGTGAACGTTGCCtttttttttt
Stachybotrys chartarum	Scha1-4	AGTATTCTCTGAGTGGTAAACGCAAAtttttttttt
*Talaromyces emersonii**	Taeme4	AGGTTGTCTGAGTGAGATTGCAttttttt
	Taeme6	CCTCGTGAACGCTGTCKTGAttttttt
*Trichophyton mentagrophytes**	Trmen3tx2-1	ACCAACGTTCCGTCAGGGTTTTATAGGGACCAACGTTCCGTCAGGGtttttttttt
	Trmen2d	GCCCCCGTCTTTGGGGGTGCGtttttttttttttttt
Trichophyton schoenleinii	Trsch2b	CGAATGGGCGCAACAAACCAttttttt
Trichophyton tonsurans	Trton1e	TCCGGCTTTCTAGGCGAtttttttttttttttt
Trichophyton rubrum	Trrub3-5	GCAGCCAATYCAGCGCCCttttttttttt
Trichophyton soudanense	Trrs1c	CAGACACCAAGAAAAAATTCTCTGtttttt
Trichophyton violaceum	Trvio1c	CAGACACCAAGGAAAATTCTCTGttttttt
Trichophyton verrucossum	Trver2e	TAGGGATCAGCGTTCCATCAtttttttt
*Trichophyton terrestre**	Trter1	AAACACGGTCTTAACTGACCATCTAGG
	Trter6b	TTAACCAAAGTCCCCCAGGtttttttttttttttt
Trichoderma viride	Tvir2-1	AACCAAACTCTTTCTGTAGTCCCCTCtttttttttt
Ulocladium consortiale	Ulcon2-3	CCAAGGTaAGCATCCACAAAGCCTT

＊A mixture of multiple probes to identify a species due to intraspecies ITS sequence variation.

＊＊A group-specific probe used to detect several closely related species.
